# The Relationship of Achievement Goal Orientations and 21st Century Skills Acquisition with the Entrepreneurship of Pre-Service Teachers

**DOI:** 10.3390/jintelligence12100097

**Published:** 2024-10-01

**Authors:** Hasan Yücel Ertem

**Affiliations:** Ereğli Faculty of Education, Zonguldak Bülent Ecevit University, Ereğli 67300, Türkiye; ertem@beun.edu.tr

**Keywords:** achievement goal orientations, skill acquisition, entrepreneurship, teacher education, higher education, pre-service teachers, 21st century skills

## Abstract

Teachers’ goal orientations and their adaptation to the 21st century determine many questions in teacher training and professional development. One of these aspects is entrepreneurship, such that the achievement goal orientations and 21st century skills of pre-service teachers have a positive role in entrepreneurship. The present study aimed to investigate the predictive role of the achievement goal orientations and 21st century skills of pre-service teachers in relation to entrepreneurship. To this end, a correlational design was conducted to analyze relationships between variables. The sample in the current study consisted of 282 pre-service teachers studying at a Faculty of Education in Anatolia Province, Turkey. The instruments were the Achievement Goal Orientations Scale, 21st Century Learning Skills Acquisition Scale, and The Scale of Entrepreneurship of College Students. The data obtained from these scales were analyzed using structural equation modeling. The results showed that the structural model of the relationships between achievement goal orientations, 21st century skills, and entrepreneurship had an adequate goodness of fit, so that most of the achievement goal orientations and 21st century skills of pre-service teachers predicted either directly or indirectly their entrepreneurship skills. The conclusion was that entrepreneurial education activities related to teacher entrepreneurship can be planned by considering 21st century skills and achievement goal orientations in teacher education.

## 1. Introduction

The future of students is shaped in schools, where teachers have the responsibility to fulfill this mission of schools. In this way, the skills of teachers become more important. In order to promote these skills, places where teacher training takes place are needed. In other words, faculties of education have the task of improving teachers’ skills. Empowering pre-service teachers at universities contributes to school effectiveness and school improvement. Investigating the skills of pre-service teachers therefore leads to positive results not only in research but also in practice.

Teachers’ goal orientations determine many questions in teacher training and professional development. Arslan (2011) stated that students’ goals in participating in educational activities influence how they engage in learning activities, how much they participate, and how much they attend. In a classroom, both students and teachers can be goal oriented. Students are oriented toward the goal of learning, while teachers are oriented toward the goal of teaching. Throndsen and Turmo (2013) explained that students’ success-oriented goal orientation can trigger teachers’ goal orientation for teaching. The pedagogical environment is influenced by the teachers’ goal orientations, so teachers shape the classroom environment to achieve their purposes. Leondari and Gialamas (2002) related teachers’ motivations to their goal orientations. Also, Liu et al. (2023) showed the role of achievement goal orientation in teacher support. In addition, the reasons why individuals determine a goal, how they achieve it, and how performance is checked are critical factors in goal orientation (Yıldızlı 2016). Church et al. (2001) emphasized that achievement goal orientations are closely related to student educational outcomes, so researchers need to focus on classroom environment variables that are critical to improving children’s orientations. Similarly, Dickhäuser et al. (2021) concluded that there is a close relationship between the motivational school climate and achievement goal orientations of teachers. To summarize, goal orientations for instruction are becoming increasingly important for a healthy learning environment.

Goal orientations are addressed within the framework of achievement goal theory, which focuses on issues of motivation and learning in the classroom (Anderman and Wolters 2005). Further, Lee and Anderman (2020) declared that goal orientations are linked to the academic development of students. Goal orientation theorists have named several types of goal orientation that are widely used in research. Dweck (1986) defined learning goal orientation and performance goal orientation. In addition, VandeWalle et al. (2001) stated a perspective on goal orientation that includes the following: proving goal orientation, avoiding goal orientation, and ability. Butler (2007) showed the dimensions of mastery, talent approach, ability avoidance, and work avoidance. In addition, Butler (2012) revised the model of goal orientation, which has a five-factor structure, by adding a relationship goal. Meece et al. (2006) explained goal structures in the classroom via goal orientations. Goal orientations in the mastery domain are related to improving one’s skills or mastering new skills, while goal orientations in the achievement domain arere related to focusing on high performance relative to others through social comparisons. In the 21st century, achievement goal orientations are mostly linked to classroom dynamics. To illustrate, Ketonen et al. (2023) stated that classroom orientation had an impact on the achievement goal orientations of students in lower secondary school. In conclusion, achievement goal orientations have significance in 21st century.

Skills in the 21st century are different from the 20th century since the skills of people for work, citizenship, and self-realization have been differentiated. The most important reason for this difference is the emergence and rapid spread of advanced information and communication technologies. In parallel with the rapid development of knowledge and communication and the rapid growth of the global economy, national and international research institutions have made great efforts to develop 21st century skills. The Partnership for 21st Century Skills (P21) (2019) classified 21st century student skills as follows: “knowledge, media and technology skills (information literacy, media literacy, and ICT literacy), learning and innovation skills (creativity and innovation, critical thinking and problem solving, communication, and collaboration), media and technology skills (information literacy, media literacy, and ICT literacy) and life and career skills (flexibility and adaptability, enterprise self-direction, social and intercultural skills, efficiency and accountability, and leadership and responsibility).” These 21st century skills were also investigated in the education field, in which researchers mostly focus on teaching 21st century skills. Almazroa and Alotaibi (2023) examined the teaching of 21st century skills within teacher education programs and concluded that initial teacher training has a critical significance.

In recent years, 21st century skills are trending topics due to skill diversity and timeliness. These skills have great significance in educating future generations. These 21st century skills have been examined in educational contexts by researchers. Larson and Miller (2011) related 21st century skills with the preparation of students for the future. Further, Kang et al. (2012) underlined the importance of 21st century skills to both adapt children for the new era and prepare suitable educational environments. In parallel to this idea, Cho (2012) focused on 21st century skills in terms of teaching methods and concluded that cultural support, leadership, school vision, and technology integration determined these skills. Similarly, 21st century skills were related to STEM education, which drew the attention of scholars in science and engineering fields (Bell et al. 2018).

Entrepreneurship can be seen as an effort to recognize, exploit and evaluate the opportunities around us, to bring them to life and thus make life more worth living. Entrepreneurship is related to productivity, which is expected to gain great importance in terms of art, science, and economic applications (Akyürek and Şahin 2013). While entrepreneurship education is defined as preparing students for the world of business (Kirby 2007), it can be broadly defined as a process in which people acquire a set of skills that can be applied in all areas of their lives. Along the way, this process brings more individual, social, and economic benefits.

In the context of globalization, entrepreneurship has become a popular term in many areas. Even though the term is often used in business and industry, it can also be found in educational science. In relation to educational science, two points are important. The first is entrepreneurship education, i.e., schools educate individuals to meet the expectations of businesses. Béchard and Toulouse (1991) explained this relationship through adult education. The authors offered a framework for entrepreneurial education based on a conformist, adaptive, transformative, and alternative pedagogical orientation. From a similar perspective, Bakar et al. (2015) linked entrepreneurship education to business and employment, explaining that people can achieve economic prosperity because of the skills acquired through entrepreneurship education. On the other hand, the second view places more emphasis on educational purposes. For example, Konaklı (2015) concluded that self-efficacy can determine social entrepreneurship in education. As mentioned in the above sections, entrepreneurship in education is seen as a 21st century skill. Further, entrepreneur education perspectives play a critical role in improving experiential learning (Motta and Galina 2023).

There is a close link between students’ achievement goal orientations and entrepreneurship. This relationship is critical in shaping the motivations and learning strategies of students. Achievement goal orientations typically refer to the reasons individuals pursue goals and how they define success. Two predominant types of achievement goals are mastery goals, which focus on self-improvement and learning, and performance goals, which emphasize demonstrating ability relative to others (Elliot and McGregor 2001). Research indicates that entrepreneurship education can positively impact mastery goal orientations. A study by Albright and Elman (2020) found that entrepreneurship programs often encourage a growth mindset, which aligns with mastery goals. By focusing on innovation and problem-solving rather than merely outperforming peers, these educational experiences foster a learning-oriented environment where students are motivated to enhance their skills and knowledge. Conversely, entrepreneurship education also interacts with performance goal orientations. According to the work of DeWitt et al. (2018), entrepreneurial settings can stimulate performance goals when students are encouraged to compete in business simulations or pitch competitions. These competitive elements can drive students to demonstrate their abilities and achieve external validation, which aligns with performance goal orientations.

Entrepreneurship education has been increasingly linked to the development of 21st century skills, a set of competencies deemed essential for success in the modern world. According to a study by Neck and Greene (2011), entrepreneurship education fosters critical thinking and problem-solving by engaging students in real-world challenges that require innovative solutions. This hands-on approach not only enhances students’ analytical abilities but also nurtures creativity, a key component of 21st century skill sets (Sullivan 2019). Moreover, entrepreneurship education is closely associated with improved collaboration and communication skills. Research by Jardim et al. (2021) highlighted that entrepreneurial perspectives in education often involve dynamics like teamwork, entrepreneurial skills, and self-confidence, which help students develop interpersonal skills and the ability to convey ideas effectively. On the other side, 21st century skills have a positive effect on entrepreneurship education. The development of the 21st century skills of pre-service teachers would increase the entrepreneurship of them. Boyles (2012) underlined the importance of 21st century skills on entrepreneurial competencies in undergraduate entrepreneurship education. In addition, Ghafar (2020) explored the idea that 21st century skills are priorities in entrepreneurship education in higher education institutions. Moreover, 21st century skills were separately linked to entrepreneurship. To illustrate, cognitive skills (Hafer and Jones 2015), autonomy (Van Gelderen 2010), collaboration (Mallett 2019), and flexibility (Yurdagul 2017) are the skills investigated in entrepreneurship education.

It can be concluded that achievement goal orientations influence positive academic achievement (Alhadabi and Karpinski 2020). However, it was found that the links between goal orientations and other educational structures and processes are insufficient. It is also noticeable that there is only a limited perspective on entrepreneurship education in schools. Even when there are gains in the educational programs, it has been found that there are deficits in terms of the educational level, content, and assessment dimensions. Despite numerous works on entrepreneurship education and its potential in the literature, there is no study on 21st century skills and achievement goal orientations in relation to entrepreneurship. For these reasons, the present study will make an important contribution to the research field.

The aim of this study is to investigate the relationship of achievement goal orientation and 21st century skills in pre-service teachers with entrepreneurship. In this context, the research questions of the current study can be listed as follows:

- Is there a significant relationship between the dimensions of both 21st century skills and achievement goal orientations and entrepreneurship?
- Do the dimensions of 21st century skills and the dimensions of achievement goal orientations in pre-service teachers show a significant relationship with entrepreneurship?

## 2. Materials and Methods

The present study is a correlation study from relational models. In correlation studies, the relationships between two or more variables are examined (Karasar 2005). In this study, the relationships between pre-service teachers’ goal orientations, 21st century learning skills, and their views on entrepreneurship were determined. The dependent or outcome variable in the study was entrepreneurship, while the independent or predictor variables in the study were pre-service teachers’ goal orientations and 21st century learning skills.

The data in the study were obtained from 282 fourth grade pre-service teachers who were studying in teaching programs of Faculty of Education at a college in an Anatolian province. The sample was randomly selected considering the strata of each teaching program. A total of 176 participants were female pre-service teachers while 106 of them were male pre-service teachers. There were 31, 66, 80, 39, and 66 participants from natural science, educational science, basic education, special education, and social science programs, respectively. In addition, the current study has three instruments. These are described as follows below:

Achievement Goal Orientations Scale: It was developed by Midgley et al. (1998) and is used to determine the goal orientation of pre-service teachers. Items of the scale are in the form of 5-Likert scale. The scale, which was adapted to the Turkish language by Akin and Çetin (2007), consists of three factors. These are the factors of learning orientation, performance orientation, and performance avoidance orientation. The internal consistency coefficients of Cronbach’s alpha for these factors were 0.77, 0.79, and 0.78. The test–retest reliability of the scale was reported as 0.95, 0.91, and 0.94 for each factor, which indicated higher reliability by considering recommendations of Hair et al. (2006) for internal consistency.

21st Century Learning Skills Usage Scale: It was developed by Orhan-Göksün (2016). Items of the scale are in the form of 5-Likert scale. It measures the degree of utilization of 21st century learning skills. This measurement tool consists of four factors. These factors were named cognitive skills, autonomous skills, collaboration skills, and flexibility and innovation skills. To test the reliability of this measurement instrument, a test–retest method was used. Valid data was collected from 30 of the participants and the Pearson correlation coefficient was calculated with this normally distributed data. Internal consistency coefficient of the scale was found to be 0.89. which indicated higher reliability by considering recommendations of Hair et al. (2006) for internal consistency. In addition, Cronbach’s Alpha Values of cognitive skills, autonomous skills, collaboration skills, and flexibility and innovation skills are 0.88, 0.71, 0.67, and 0.82, respectively.

The Scale of Entrepreneurship of College Students: It was developed by Yılmaz and Sünbül (2009) to determine the entrepreneurial characteristics of students. Items for the scale are in the form of 5-Likert scale. There are no sub-factors in this scale. Before preparing the items for the scale, a total of 90 undergraduate students wrote essays on entrepreneurship. The students’ written opinions, ideas, feelings, and thoughts were transformed into sentences and items. The data was subjected to a factor analysis (validity analysis) and a Cronbach’s alpha reliability analysis. Cronbach Alpha coefficient was found to be 0.90, which indicated higher reliability by considering recommendations of Hair et al. (2006) for internal consistency.

In the data analysis, arithmetic means and standard deviations were compared within the framework of descriptive statistics. Inferential statistics were used to examine the relationships between goal orientations, 21st century learning skills, and entrepreneurship. To uncover these relationships, a Pearson moments multiplicative correlation was performed. In addition, structural equation modeling (SEM) was conducted to examine the causal effects of goal orientations and 21st century skills on entrepreneurship. The reason why SEM was chosen instead of other statistical procedures was because the purpose was testing a theoretical causal model. The model was tested in four steps according to Kline (2011). In the first step, model specification was conducted by generating a hypothesis for a structural model. In the second step, model identification involved the statistical program providing an estimate for the parameters of the model. The third step, model estimation, was performed by comparing the hypothesized model with the observed model in the statistical program. Finally, the model evaluation was performed by assessing the model using some goodness-of-fit indices. For both confirmatory factor analysis and structural equation modeling, the χ^2^/df, RMSEA, NFI, NNFI, and CFI criteria were considered. The assumptions were taken into account for all analyses.

Before the main analysis, the prerequisites and assumptions had to be checked. The adequacy of the sample size is a prerequisite, so Kline (2011) suggested a minimum number of 200 subjects to conduct the SEM. This requirement was met with a sample size of more than 200. According to Field (2009), the assumptions of correlation analyses are normality, homoscedasticity, linearity, independence of errors, absence of multicollinearity, and influential observations. Assumption of normality was assumed by checking P–P plots and shapes of the histogram while homoscedasticity was assumed by considering scatter plot. In addition, linearity was checked by considering partial regression plots and assumed. According to Durbin and Watson (1951), coefficient value must be between 1.00 and 3.00 for the errors to be unrelated. The 1.91 value of Durbin–Watson proved independency of errors. Influential observations were checked through Cook’s distance and DFbeta values. Cook and Weisber (1982; as cited in Field 2009) recommended that Cook’s distance (a measure of the influence of case) must be smaller than 1.00. Cook’s distance in the current study had values changing between 0 and 0.4 so this criterion was provided. Tabachnick and Fidell (2007) stated DFbeta values should be smaller than 1.00. Statistical package output showed that values for DFbeta are smaller than 1.00 so that this criterion was also confirmed. As the last assumption, Tolerance and Variance Influence Factor (VIF) values were checked for the absence of multicollinearity. Myers (1990; as cited in Field 2009) recommended that Tolerance values must be larger than 0.10 while VIF values must be lower than 10. The analysis output indicated that while Tolerance values changed between 0.61 and 0.72, VIF values varied from 1.39 to 1.65, so multicollinearity is absent.

## 3. Results

Considering the descriptive statistics, the means and standard deviation values of the variables were calculated. These values are represented in Table 1.

Among the variables, the cognitive skills of pre-service teachers (M = 4.06, SD = 0.46) seem to be more positive than the innovative skills (M = 3.87, SD = 0.78), entrepreneur skills (M = 3.87, SD = 0.43), autonomous skills (M = 3.68, SD = 0.56), collaborative and flexible skills (M = 3.53, SD = 0.62), learning skills (M = 3.52, SD = 0.62), performance approach (M = 3.45, SD = 0.90), and performance avoidance (M = 2.47, SD = 1.01) of pre-service teachers.

To examine the relationships among the variables, a Pearson correlation was performed. The findings revealing the relationship between the goal orientations, 21st century learning skills, and entrepreneurship levels of pre-service teachers are demonstrated in Table 2.

While the interrelationships among the variables were interpreted, the *p* values of the relationships were also interpreted. According to Gravetter and Wallnau (2013), *p* values indicate the level of statistically significant relationships within a hypothesis, in which a *p* value close to 0 shows that the difference or effect is caused by a factor rather than chance. When Table 2 was examined, the entrepreneurship scores had a relationship with innovation (r = 0.43; *p* < .01), collaboration and flexibility (r = 0.47; *p* < .01), autonomy (r = 0.48; *p* < .05), learning (r = 0.48; *p* < .01), cognition (r = 0.71; *p* < .01), and performance approach (r = 0.13; *p* < .05). All these relationships were positive, such that an increase in one would lead to an increase in the others. Further, the relationship of entrepreneurship with 21st century learning skills was more powerful than that with goal orientations. On the other side, all variables were interrelated to each other except performance avoidance. However, performance avoidance was only related to cognition in a negative direction.

To seek an answer for the second research question, structural equation modelling (SEM) was run in a package program. The estimation method of “Weighted Least Squares” with an Asymptotic Covariance Matrix was used in the structural model. The structural model showed a high fit (χ^2^/df = 1.59, RMSEA = 0.047, NFI = 0.973, GFI = 0.983, CFI = 0.989), according to the recommendations of Hu and Bentler (1999), Tabachnick and Fidell (2007), and Wheaton et al. (1977). In addition, the values of the standardized estimates changed between 0.11 and 0.54, which are acceptable for the model goodness of fit.

**Figure 1:**
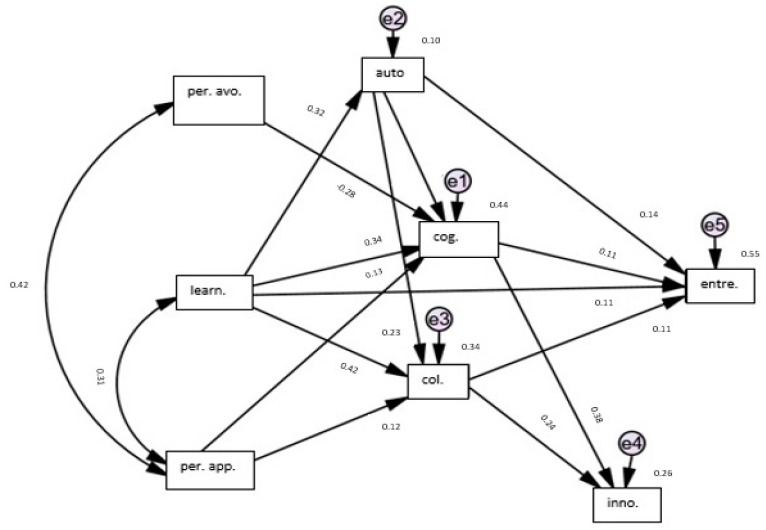
Structural model. Abbreviations: per. avo.—performance avoidance; per. app.—performance approach; learn.—learning; auto.—autonomy; cog.—cognition; col.—collaboration and flexibility; inno.—innovation; entre.—entrepreneurship.

The structural model showed that entrepreneurship was predicted by both the goal orientations and 21st century learning skills of pre-service teachers. Learning as a dimension of goal orientations had a direct effect on entrepreneurship. Moreover, it had an indirect effect on entrepreneurship, over autonomy, cognition, and collaboration and flexibility. Performance avoidance had an indirect effect on entrepreneurship over only cognition while the performance approach had an indirect effect on entrepreneurship over both collaboration and flexibility and cognition. Except for innovation, all of the 21st-century learning skills had direct effects on entrepreneurship. Further, autonomy had an indirect effect on entrepreneurship. All these relationships can be seen in Figure 1.

When the effects of paths in the analysis were examined, it was concluded that cognition (β = 0.54, *p* < .01) had the greatest direct effect on entrepreneurship while learning (β = 0.54, *p* < .01) had the greatest indirect effect on entrepreneurship. Considering the total effects, cognition (β = 0.54, *p* < .01), learning (β = 0.46, *p* < .01), autonomy (β = 0.37, *p* < .01), performance avoidance (β = −0.15, *p* < .01), collaboration and flexibility (β = 0.11, *p* < .05), and performance approach (β = 0.09, *p* < .05) had effects on entrepreneurship. All these variables explained the 55% variance in entrepreneurship.

The path analysis showed that cognition (β = 0.38, *p* < .01) had the greatest direct effect on innovation while autonomy (β = 0.19, *p* < .01) had the greatest indirect effect on innovation. Considering the total effects, cognition (β = 0.38, *p* < .01), collaboration and flexibility (β = 0.24, *p* < .01), autonomy (β = 0.19, *p* < .01), performance avoidance (β = −0.10, *p* < .05), and performance approach (β = 0.08, *p* < .05) had effects on innovation. All these variables explained the 26% variance in innovation.

In the path analysis, learning (β = 0.49, *p* < .01) was found to be the variable showing the greatest total effect on collaboration and flexibility. Further, autonomy (β = 0.23, *p* < .01) and performance approach (β = −0.12, *p* < .05) showed direct effects on collaboration and flexibility. These values explained the 34% variance in collaboration and flexibility.

Considering the effects on cognition, learning (β = 0.46, *p* < .01) had the greatest total effect while autonomy (β = 0.37, *p* < .01) had the greatest direct effect. Moreover, performance avoidance (β = −0.28, *p* < .01) and performance approach (β = 0.13, *p* < .01) showed direct effects on cognition. Autonomy, learning, performance avoidance, and performance approach explained the 44% variance in cognition.

Finally, autonomy was affected by only learning (β = 0.32, *p* < .01), such that learning explained the 10% variance in autonomy. Table 3 summarizes all these direct, indirect, and total effects, in addition to the squared multiple correlations.

## 4. Discussion

The results of this study show that the achievement goal orientations, 21st century skills, and entrepreneurship of pre-service teachers are interrelated. In addition, the 21st century skills, including cognition, autonomy, collaboration and flexibility, and innovation, and achievement goal orientations, including performance avoidance, performance approach, and learning orientations, had either a direct or indirect influence on entrepreneurship. Furthermore, the 21st century skills were influenced either by themselves or by achievement goal orientations. To summarize, achievement goal orientations and 21st century skills in pre-service teachers influence their entrepreneurship.

The picture painted by the current study on the relationship between achievement goal orientations, 21st century skills, and entrepreneurship offers perspectives that are consistent with the literature. Orhan-Göksün (2016) examined 21st century skills in her dissertation and concluded that 21st century learners need to be able to multitask in order to achieve their goals. In this regard, cognitive skills, autonomous skills, skills based on collaboration and flexibility, and innovation skills can be associated with entrepreneurship. On the other hand, Ghafar (2020) investigated the relationship between 21st century skills and entrepreneurship in higher education and concluded that entrepreneurship education is more effective when 21st century skills are integrated. Boyles (2012) investigated the relationships between 21st century knowledge, skills, abilities, and entrepreneurial competencies. The author found that cognitive, social, and action-oriented entrepreneurship are linked to 21st century knowledge, skills, and abilities, including literacy, independent thinking, communication, and productivity.

The current study has shown that 21st century capabilities, including autonomy, cognition, innovation and collaboration, and flexibility, are influenced by goal orientations. There are many studies in the literature that deal with this type of relationship. In this respect, the current study is consistent with the other studies in the literature. Coutinho and Neuman (2008) presented a model on metacognition, learning style, learning goal orientation, and self-efficacy. The authors found that self-efficacy was the strongest predictor of performance, while self-efficacy was predicted by mastery intention, mastery avoidance, performance intention, and performance avoidance. Dimensions of 21st century skills such as collaboration and flexibility reflect the climate in the classroom. Ames and Archer (1988) examined the analysis of classroom climate using achievement goals and concluded that a focus on mastery goals promotes student learning. In addition, González-Pérez and Ramírez-Montoya (2022) examined the components of education 4.0 by moving from industry 4.0 and stated that educational perspectives based on social needs, competencies, and systemic thinking play an important role in improving 21st century skills. From a similar angle, Kennedy and Sundberg (2020) declared that teaching 21st century skills to students is required to prepare students for a digital future.

The result of the current study is consistent with the idea in the literature that goal orientations have an influence on entrepreneurship. Brunel (1999) investigated the relationship between performance goal orientations and motivation and found that a mastery-oriented climate is related to intrinsic motivation. This relationship can be explained with entrepreneurship as the logic of entrepreneurial action is based on motivations and attitudes (Estay et al. 2013). Arslan (2011) conducted a study to investigate the goal orientations of pre-service teachers and their views on constructivism. The author found that there is a significant relationship between goal orientations and constructivism. Constructivist approaches are modern pedagogical approaches that place learners at the center and encourage entrepreneurship. Similarly, Culbertson et al. (2011) conducted a study to examine factors that influence entrepreneurship, such that learning goal orientations and performance goal orientations predicted entrepreneurship in college students.

In summary, 21st century skills were found to be predictors of entrepreneurship in the current study. This result is consistent with the literature. Leitch and Harrison (1999) proposed a process-oriented approach to entrepreneurship education. The authors emphasized that entrepreneurship is influenced by learning, communication, leadership, and management of schools that are closely linked to 21st century skills. Pache and Chowdhury (2012) investigated social entrepreneurship education and concluded that a combination of several logics such as collaboration, fairness, and transparency is crucial for the development of institutionally embedded entrepreneurs. On the other hand, entrepreneurship is also associated with the 21st century skills framework. Obschonka et al. (2017) focused on entrepreneurship as a 21st century skill. The authors found that entrepreneurship readiness and career intentions were predicted by a number of entrepreneurial competencies, including leadership, self-esteem, creativity, and proactive motivation, which may be related to 21st century skills. In conclusion, the idea in the literature that entrepreneurship is closely related to 21st century skills was confirmed by the current study.

The present study has implications when looking at the results. In terms of research, the study contributed to the literature as it attempted to fill a gap in the literature by offering a model based on the relationships between goal orientations, 21st century learning capabilities and entrepreneurship. In theoretical terms, the results of this study can enrich the theoretical discussion in the field of teacher education. In terms of practice, policy makers at the Ministry of Education and higher education institutions could develop policies to promote entrepreneurship among pre-service teachers. This issue points to a significant interaction problem between schools and higher education institutions. As emphasized in the literature (Demir and Çamlı 2011; Kocadere and Aşkar 2013; Saka 2019), there is a communication and cooperation problem between schools and higher education institutions. In this regard, a bridge could be built between these institutions to improve the skills and knowledge of pre-service teachers.

The researchers of the current study offer some issues to researchers and practitioners. First, because the study was conducted with a smaller sample and a quantitative method, the researchers recommend similar studies with larger samples and qualitative or mixed methods to gather more detailed and valid information about the phenomenon. Despite the potential for generalization to the population due to the random sample, the results are not transferable to other contexts. In other words, the study lacks external and ecological generalizability. External generalizability is the generalization of the findings of a study to other settings while ecological generalizability is the generalization of the findings of a study to real-life situations (Creswell and Creswell 2021). The findings of the current study may not be valid in a metropolitan context. Therefore, it is recommended that further research be conducted in different contexts such as different regions, different cities, and different schools. In addition, researchers could focus on other antecedents of entrepreneurship in larger contexts in the future. The final recommendations are aimed at policy makers and practitioners. Policy makers could take legitimate measures to improve the entrepreneurship of pre-service teachers. For example, the curricula of faculties of education could be revised by focusing on goal orientations, 21st century learning skills, and the entrepreneurial nature of teaching. With this revision, the practice of beginning teachers could be reorganized and improved. College administrators and the teacher education departments of the Ministry of Education could work together to develop teachers in many areas like leadership, instructional technology, and financial literacy.

## Figures and Tables

**Table 1:** Descriptive statistics.

Variables	Mean	Standard Deviation
Entrepreneurship	3.87	0.43
Learning	3.52	0.62
Performance approach	3.45	0.90
Performance avoidance	2.47	1.01
Cognition	4.06	0.46
Innovation	3.87	0.78
Autonomy	3.68	0.56
Collaboration and Flexibility	3.53	0.62

**Table 2:** Correlation matrix.

Factors	1	2	3	4	5	6	7	8
1. Innovation	1.00							
2. Collaboration and flexibility	0.41 **	1.00						
3. Autonomy	0.30 **	0.38 **	1.00					
4. Cognition	0.48 **	0.45 **	0.48 **	1.00				
5. Performance avoidance	−0.03	0.11	0.07	−0.18 **	1.00			
6. Performance approach	0.11	0.29 **	0.16 **	0.18 **	0.43 **	1.00		
7. Learning	0.34 **	0.53 **	0.32 **	0.50 **	0.04	0.32 **	1.00	
8. Entrepreneurship	0.43 **	0.47 **	0.48 **	0.71 **	−0.10	0.13 *	0.48 **	1.00

** *p* < 0.01; * *p* < 0.05.

**Table 3:** Effect table of path analysis.

Effect	Direct	Indirect	Total	R^2^
**On autonomy**				0.10
Learning	0.32	-	0.32	
**On cognition**				0.44
Autonomy	0.37	-	0.37	
Performance avoidance	−0.28	-	−0.28	
Achievement performance	0.13	-	0.13	
Learning	0.34	0.12	0.46	
**On collaboration and flexibility**				0.34
Autonomy	0.23	-	0.23	
Performance approach	0.12	-	0.12	
Learning	0.42	0.07	0.49	
**On innovation**				0.26
Collaboration and flexibility	0.24	-	0.24	
Cognition	0.38	-	0.38	
Performance avoidance	-	−0.10	−0.10	
Performance approach	-	0.08	0.08	
Autonomy	-	0.19	0.19	
**On entrepreneurship**				0.55
Autonomy	0.14	0.23	0.37	
Cognition	0.54	-	0.54	
Collaboration and flexibility	0.11	-	0.11	
Learning	0.11	0.35	0.46	
Performance avoidance	-	−0.15	−0.15	
Performance approach	-	0.09	0.09	

## Data Availability

The dataset analyzed here might be available upon request from interested researchers.
